# Supplementary material for: Word learning from a tablet app: Toddlers perform better in a passive context
Source: PLoS One. 2020 Dec 1;15(12):e0240519. doi: 10.1371/journal.pone.0240519 (PMC7707543; doi:10.1371/journal.pone.0240519)
Supplement: S1 Appendix — (DOCX) [file pone.0240519.s001.docx]

# S1 Appendix

**Phonotactic rules and constraints of German**

All pseudowords used in the study follow the phonotactic rules and constraints of German. All words used in the study are bisyllabic and stressed on the first syllable:

[ˈbat͜ʃa], [ˈfoːma], [ˈkoːlat], [ˈvidɛk͜s]

As outlined in [42], German syllables consist of a consonant onset, a vocalic nucleus and a consonant coda. However, only the nuclear vowel is obligatory, so that an empty coda as in the first syllable of *Kolat* and the second syllable of *Batscha* and *Foma* is acceptable. The consonant clusters used in the pseudowords are also common in words that toddlers encounter in their everyday lexical environment: [t͜ʃ] appears in words like *Rutsche* ‘slide’ or *Matsch* ‘mud’, while [k͜s] appears in *Hexe* ‘witch’ and *sechs* ‘six’.

**Phonotactic rules and constraints of Malay**

All pseudowords used in the study follow the phonotactic rules and constraints of Malay. All words used in the study are bisyllabic with no lexical stresses but instead with a rise-fall pitch movement (where its start is indicated by [ˈ]):

[ˈbanuŋ], [ˈifi], [ˈmipo], [ˈpafka]

In general, the Malay accent lacks stress [43] and a majority of the Malay lexicon is based on disyllabic root morphemes [44]. As outlined in [45], syllables have the C_1_VC_2_ structure, where both C_1_ and C_2_ are optional consonants and V is a monophthong. Thus, the syllables making up the words *banung*, *ifi*, and *pafka* are valid. While in the native lexis, only /i/, /u/, and /a/ are allowed in final open syllable [45], /o/ in *mipo* is also found in loanwords like *solo* ‘solo’ and *koko* ‘cocoa’.

[42] Van Oostendorp, M. (2020): Germanic Syllable Structure. In M.T. Putnam & B.R. Page: *The Cambridge Handbook of Germanic Linguistics* (33-48). Cambridge University Press.

[43] Mohd Don, Z., Knowles, G., & Yong, J. (2008). How words can be misleading: a study of syllable timing and “stress” in Malay. *Linguistics Journal*, *3*(2).

[44] Adelaar, K. A. (1992). *Proto Malayic: The reconstruction of its phonology and parts of its lexicon and morphology*. Dept. of Linguistics, Research School of Pacific Studies, the Australian National University.

[45] Clynes, A., & Deterding, D. (2011). Standard Malay (Brunei). *Journal of the International Phonetic Association*, *41*(2), 259-268.
