# Supplementary material for: Word learning from a tablet app: Toddlers perform better in a passive context
Source: PLoS One. 2020 Dec 1;15(12):e0240519. doi: 10.1371/journal.pone.0240519 (PMC7707543; doi:10.1371/journal.pone.0240519)

# S2 Appendix

**Fig. B1: Reaction time by trial.** Reaction time for correct responses in the familiar, 2-AFC and 4-AFC test phases, split by age group and condition, with outliers (> 2 SD) removed.


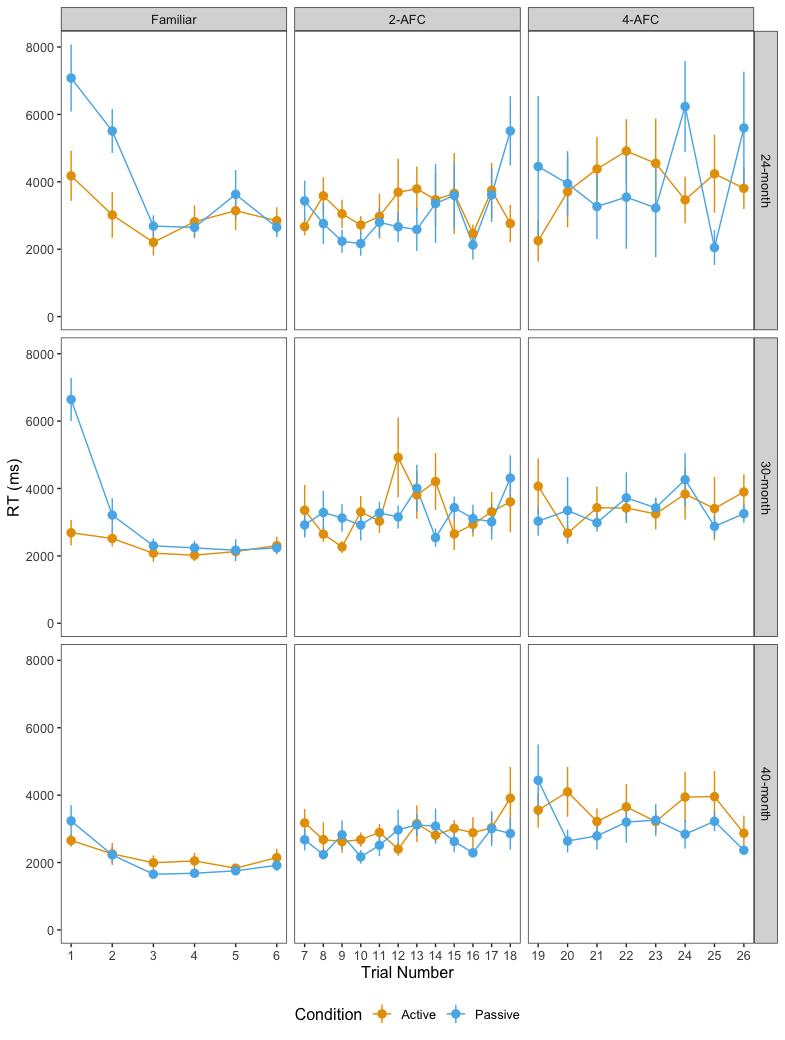


**Fig. B2: Accuracy by trial.** Accuracy in the familiar, 2-AFC and 4-AFC test phases, split by condition. Dashed line represents chance (.5) in the familiar and 2-AFC test phases, dotted line represents chance (.25) in the 4-AFC test phase.


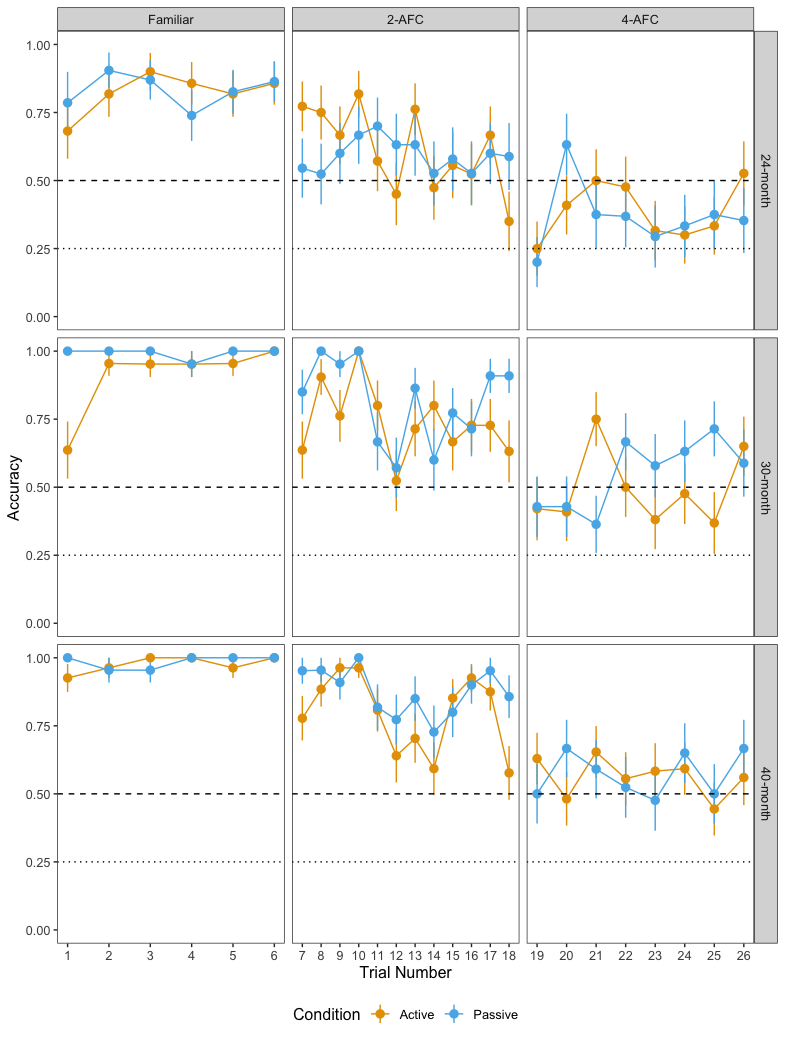

Supplement: S2 Appendix — (DOCX) [file pone.0240519.s002.docx]
