# Supplementary material for: Word learning from a tablet app: Toddlers perform better in a passive context
Source: PLoS One. 2020 Dec 1;15(12):e0240519. doi: 10.1371/journal.pone.0240519 (PMC7707543; doi:10.1371/journal.pone.0240519)
Supplement: S3 Appendix — (DOCX) [file pone.0240519.s003.docx]

S3 Appendix

In addition to the intercept-only models reported above, we also ran models with a maximal random effects structure for both experiments.

The models for Experiment 1 include by-participant-pair adjustments to the effect of condition as well as by-object adjustments to the effect of the interaction between condition and age, resulting in the following model structures:

RT_log_ ~ (Condition * Age) + (1 + Condition | Participant pair) + (Condition * Age | Object)

Accuracy ~ (Condition * Age) + (1 + Condition | Participant pair) + (Condition * Age | Object)

Results for the RT models are detailed in Table C1. Breaking the data by age, we found a significant effect of condition at 24-months (β = 0.184, p = .034) and 30-months (β = 0.114, p < .001) in the familiar trials, with children from both age groups responding faster when they were assigned to the active condition. No such effect of condition was found in the 40-month age group (β = -0.018, p = .645). With regard to the 2-AFC and 4-AFC trials, no such effect of condition was found across all age groups.

Results for the accuracy models are detailed in Table C2. With regard to the familiar trials, we found a significant effect of condition overall, although this interacted with age highlighting a difference in the effect of condition at 24-months and 40-months. Breaking the data by age, a significant effect of condition was found at 30-months (β = 8.274, p < .001), with children being more accurate in the passive condition, but no such effect was found at 24-months (β = -0.059, p = .788) and 40-months (β = 2.724, p = .326) in the familiar trials. With regard to the 2-AFC trials, again we found a main effect of condition across all ages which did not interact with age. Nevertheless, exploratory analyses examined the effect of condition at the different ages (24-months: (β = -0.049, p = .688; 30-months: β = 0.288, p = .055; 40-months: β = 0.340, p = .018). With regard to the 4-AFC trials, no such effect of condition was found across all age groups.

Table C1. Experiment 1: Estimates for the reaction time model with a full random effects structure.

|  | Familiar | | | 2-AFC | | | 4-AFC | | |
| --- | --- | --- | --- | --- | --- | --- | --- | --- | --- |
|  | β | SE | p | β | SE | p | β | SE | p |
| (Intercept) | **7.732** | **0.020** | **<.001** | **7.848** | **0.028** | **<.001** | **7.990** | **0.058** | **<.001** |
| Condition | **0.088** | **0.036** | **.015** | -0.039 | 0.036 | .267 | -0.041 | 0.034 | .233 |
| Age - 30m | 0.017 | 0.051 | .742 | **0.090** | **0.040** | **.022** | 0.037 | 0.074 | .618 |
| Age - 40m | **-0.166** | **0.044** | **<.001** | -0.017 | 0.038 | .664 | 0.001 | 0.070 | .990 |
| Condition* Age - 30m | 0.027 | 0.042 | .515 | 0.045 | 0.048 | .344 | 0.036 | 0.051 | .481 |
| Condition* Age - 40m | **-0.105** | **0.044** | **0.016** | 0.004 | 0.049 | .932 | -0.030 | 0.045 | .503 |

Table C2. Experiment 1: Estimates for the accuracy model with a full random effects structure.

|  | Familiar | | | 2-AFC | | | 4-AFC | | |
| --- | --- | --- | --- | --- | --- | --- | --- | --- | --- |
|  | β | SE | p | β | SE | p | β | SE | p |
| (Intercept) | **3.457** | **0.429** | **<.001** | **1.224** | **0.100** | **<.001** | -0.034 | 0.123 | .782 |
| Condition | 0.567 | 0.438 | .196 | **0.199** | **0.079** | **.012** | 0.027 | 0.086 | .755 |
| Age - 30m | 0.713 | 0.769 | .354 | 0.213 | 0.145 | .141 | 0.138 | 0.129 | .284 |
| Age - 40m | 0.987 | 0.575 | .086 | **0.575** | **0.154** | **<.001** | **0.357** | **0.127** | **.005** |
| Condition* Age - 30m | 1.027 | 0.699 | .142 | 0.113 | 0.119 | 0.344 | 0.097 | 0.128 | .446 |
| Condition* Age - 40m | -0.472 | 0.496 | .341 | 0.140 | 0.123 | 0.256 | -0.018 | 0.132 | .893 |

The models for Experiment 2 include by-participant-pair and by-object adjustments to the effect of condition, resulting in the following model structures:

RT_log_ ~ Condition + Learning looks + (1 + Condition | Participant pair) + (1 + Condition | Object)

Accuracy ~ Condition + Learning looks + (1 + Condition | Participant pair) + (1 + Condition | Object)

Results for the models are detailed in Tables C3 and C4.

Table C3. Experiment 2: Estimates for the reaction time model with a full random effects structure.

|  | Familiar | | | 2-AFC | | | 4-AFC | | |
| --- | --- | --- | --- | --- | --- | --- | --- | --- | --- |
|  | β | SE | p | β | SE | p | β | SE | p |
| (Intercept) | **8.072** | **0.123** | **<.001** | **8.214** | **0.151** | **<.001** | **9.252** | **0.290** | **<0.001** |
| Condition | 0.064 | 0.099 | .514 | 0.069 | 0.107 | .521 | 0.131 | 0.162 | .418 |
| Learning looks | - | - | - | -0.144 | 0.191 | .449 | **-1.555** | **0.363** | **<.001** |

Table C4. Experiment 2: Estimates for the accuracy model with a full random effects structure.

|  | Familiar | | | 2-AFC | | | 4-AFC | | |
| --- | --- | --- | --- | --- | --- | --- | --- | --- | --- |
|  | β | SE | p | β | SE | p | β | SE | p |
| Intercept | **2.929** | **0.502** | **<.001** | **0.989** | **0.393** | **.012** | **-1.220** | **0.500** | **.015** |
| Condition | 0.486 | 0.525 | .354 | **0.246** | **0.115** | **.032** | **0.290** | **0.142** | **.041** |
| Learning looks | - | - | - | -0.549 | 0.531 | .302 | 1.035 | 0.666 | .120 |
