# Supplementary material for: Word learning from a tablet app: Toddlers perform better in a passive context
Source: PLoS One. 2020 Dec 1;15(12):e0240519. doi: 10.1371/journal.pone.0240519 (PMC7707543; doi:10.1371/journal.pone.0240519)
Supplement: Attachment — Submitted filename: Response to Reviewers Ackermann Lo.docx [file pone.0240519.s004.docx]

As indicated by Reviewer 1, there are different aspects to active learning making a stronger theoretical justification of the particular choice of activity (tapping to select items for learning) implemented here necessary.

Furthermore, speculation about what underpins the benefit observed in the passive group needs be constrained through further investigation, as Reviewer 1 points out. Which aspects of your study are germane to active learning and which ones are artefacts of the methodology? In the Discussion, you mention a range of explanations for why tapping may have caused a detrimental effect in the active group (draining resources, having dual function of indicating preference vs. comprehension, prepotent behaviour). Both reviewers also identify other confounds such as potential differences in the pragmatics of the instruction, and differences in the degree of novelty and function of tapping between groups that should be ruled out in a second experiment.

Although I fully appreciate the practical problems associated with testing small children I also share Reviewer 1’s concern about the limited sample size, especially given the amount of fixed effects in your models. In addition to this, further information and possible revision of the statistical models is required (e.g. Did you use a logit link function for the accuracy data? Did you include random slopes [i.e. a maximal random effect structure] and if not why not?)

- We have now provided more information about the model, clearly specifying the model used. As we note below, we have also now tested several more children (n=132 in Experiment 1, n=38 in Experiment 2), thereby addressing the issue of limited sample size. We have also removed the fixed effects of test.order and gender as requested by Reviewer 1.

In short, a major revision should involve one or several further experiments to clarify further what accounts for the inferior performance of the active group.

- We have now tested several more children (132 children in Experiment 1 and 38 children in Experiment 2) to examine the developmental time course of the passive benefit we reported in the earlier manuscript (new Experiment 1) as well as to examine a more implicit measure of recognition performance (new Experiment 2). These studies suggest that there is no evidence for differences in the learned competence of children assigned to the active or the passive condition. Both active and passive children recognized the target object successfully and fixated the target object similarly as indexed by the gaze data collected. However, we replicate the performance deficit, with active children showing poorer accuracy of target recognition in the tapping task, across all children tested, regardless of age or cultural background. Taken together, we suggest that these results suggest caution in advocating for an active or a passive boost in digital learning tasks.

Reviewers' comments:

Reviewer #1:

I would also prefer to see the authors conduct a follow-up study exploring the possibilities they mention in the Discussion. It is indeed possible that the observed results were driven by the novelty of the tapping action or children’s misinterpretation of the task. I think that a study that tests these conjectures would strengthen the authors’ claim and provide additional information about the factors driving children’s learning from interactive tablet apps.

- See response to Editor above. We have now included a second experiment which includes implicit gaze data to examine the extent to which the passive boost found in Experiment 1 was related to a competence deficit in children (active children did not learn the novel word-object associations as well as the passive children) or a performance deficit (active children were carried away by the thrill of tapping on the screen). We found no evidence for a competence deficit and suggest that the results are best explained by a performance deficit. Taken together, we suggest that these results suggest caution in advocating for an active or a passive boost in digital learning tasks.

Specific concerns and other comments:

Line 25-26: Either “by” or “due” for both alternatives would sound better

- Done

Line 28: that’s a “yoked” design, correct? Perhaps mention this to orient the reader

- Done

Broad comment: I think that a distinction should be made between different types of “active” learning. One way to make an app more interactive is by delegating the choice of an image to be labelled to the player. Another way is by introducing an element of (pseudo) socially contingent exchange (e.g., voice feedback to the child’s actions, or a video of a speaker reacting to such actions). There might be differences in learning outcomes, depending on how the “active” learning component is implemented. I suggest including this point in the GD.

Line 63: I see that the idea is to see whether any sort of contingent feedback would work (given that a manipulation of this sort showed an effect before), but I would still suggest mentioning the difference between social and non-social feedback

- We have now added explicit reference to social feedback, pseudo-social contingent feedback and active learning with regards to allowing children to choose the kinds of information they want to learn in the Introduction.

Line 80: the SES effect does not seem particularly relevant to the narrative; I suggest either dropping this line or explaining the relevance of this finding (e.g., lower-SES children may have a different pattern of attention distribution, or different access to tablet devices, or something else).

- We have now removed this sentence from the manuscript

Lines 77-83: No need to cite again, it’s clear that the same study is being referenced throughout the paragraph (same goes to other paragraphs).

- Done

General suggestion about the intro: I think the authors are trying to be careful and diligent with citing earlier research but it’s making the intro excessively long. I suggest that many non-critical details about earlier studies can be dropped.

- We have now attempted to remove all excessive details about studies while citing earlier research as extensively as possible.

Line 114: It’s better to keep the labeling of conditions consistent. In the abstract the children in the “yoked” condition are referred to as “passive” participants (also, see Line 123). I suggest mentioning that a yoked design was used and then refer to the conditions as “active” and “passive” respectively.

- We have now changed this to keep consistent. We first mention that it is a yoked design and then continue to only use active and passive respectively.

Lines 121-122: What is the prediction for the active group?

- We have now outlined the predictions for the active and passive groups across all the age-groups tested.

Line 140: It would be nice to cite some vocabulary development norms. I am sure that most 30-month-olds know the names of all objects used in the study, but it would be better to rely on some formal evidence.

- We have now included data from German vocabulary norms that over 75% of 24-month-olds and close to 100% of 30-month-olds produce the familiar words tested. While German norms do not assess receptive competence, it is therefore likely that the children tested in the current study are familiar with the words presented in the familiar trials.

Line 143: It would be good to see a brief description of such phonotactic constraints. Perhaps, in an Appendix?

- We have now added an appendix that provides phonetic transcriptions of all pseudowords and how they confirm to the phonotactic rules of German and for Experiment 2, Malay.

Line 164: Did all children stay engaged with the app the entire time? If not, then the differences in the level of engagement between the active and the passive participants should be mentioned (e.g., the passive participants could have found the game less fun and looked away more often).

- We have now added a separate study that allows us to examine this in more detail. In the first study, we did not record looking time data which would allow us to examine the times spent by active and passive children looking at the trials. In the second study, however, we examined the amount of time that children spent looking at the object during the learning phase across conditions. We found that passive participants looked at the target significantly more than active participants at a later window in the learning trial (from 7200ms to 9800ms). To account for this difference, we entered looking time as the target during learning as a predictor of performance at test to account for any potential differences in recognition performance across conditions. This predictor did not explain variability in the recognition performance, suggesting that even though there were differences in the looking times to target (albeit very late in the trial), this looking time difference did not influence recognition performance across conditions.

Line 180: N of participants on the pilot trials?

- We have now added information of the number of participants in the pilot test. We tested 42 children in this earlier task and realized we need to include a familiar phase to allow passive participants to practice tapping the screen.

Line 183: Please include the carrier phrase used

- Done.

Line 191: Remove the superscript in “was”?

- Done

Line 208: How many outliers were there in each condition? Any age-related patterns?

- We now report the outliers split by condition and age-group.

Line 220: It seems to be a lot of variables for a sample of 34 children (the same goes to the analyses of accuracy), I wonder if it would make more sense to run preliminary analyses for variables such as age, gender, and trial order and to only include the variables that do show significant effects to the subsequent analyses. Also, I am not sure why gender but not age is included as a variable. The authors talked about the significance of age for children’s ability to benefit from interactive apps and did not highlight the role of gender. Perhaps, it would make sense to split the sample by age (i.e., “younger half” & “older half”) and test this effect instead of gender.

- We have now increased the sample size to 132 children with 44 children in each age-group. In addition, we have run the models without the variables test.order and gender report these results. We also include age as suggested by the reviewer to the model and indeed do find an interaction between Condition (active.passive) and age in the 2-AFC task which we break down further. We thank the reviewer for this suggestion.

Line 287: I agree. I wonder if it would make sense to run a follow-up, in which children are given some practice for ~7 days prior to the study so that they do not have to master a new skill along with learning or be too carried away with the thrill of tapping on the screen.

- We have now included a second experiment which includes implicit gaze data to examine the extent to which the passive boost found in Experiment 1 was related to a competence deficit in children (active children did not learn the novel word-object associations as well as the passive children) or a performance deficit (active children were carried away by the thrill of tapping on the screen). We found no evidence for a competence deficit and suggest that the results are best explained by a performance deficit. Taken together, we suggest that these results suggest caution in advocating for an active or a passive boost in digital learning tasks.

Line 299: It reads as though the children were the ones who did not find the effect of condition. Consider rephrasing (e.g., “Despite . . . , we observed that . . .”).

Based on your recommendation we have now removed trial order from the model and have consequently removed this passage.

Reviewer #2:

Having said that, I think the study needs further justification or clarification regarding potential confounding factors (prompt, familiarization trial). First, in the active condition, the prompt was given as a form of “instruction” (i.e., "Look, here are two pictures. You can tap on one", "Tap on an object, then you’ll hear its name"). On the other hand, in the passive condition, the prompt was given as a form of “question” (i.e., “Do you see the two pictures? Are they beautiful?”). Existing evidence suggests that toddlers appear to utilize social cues such as referential intent of speakers for word learning (Bloom, 2000; Golinkoff & Hirsh-Pasek, 2006). For example, Luchkina, Sobel, & Morgan (2016) showed that 18-month-olds are sensitive to the difference between making statements and asking questions in learning novel labels. Research with preschoolers has shown that explicit instruction might help immediate learning but limit further exploration compared to pedagogical questioning (Bonawitz et al., 2011; Jean et al., 2019). I think further justification is required to conclude that the difference in word learning is due to interactivity rather than the difference in children’s inference of speaker intentions.

- We have now included a second experiment which includes implicit gaze data to examine the extent to which the passive boost found in Experiment 1 was related to a competence deficit in children (active children did not learn the novel word-object associations as well as the passive children) or a performance deficit (active children were carried away by the thrill of tapping on the screen). We found no evidence for a competence deficit and suggest that the results are best explained by a performance deficit, i.e., due to the interactivity of the task.

Second, both active and passive groups were allowed to interact with the screen during the familiarization phase, which occurred right before the 2 and 4- AFC testing phases. Thus, it is not yet clear whether the active group’s lower performance in testing was due to their active selection during learning or the relatively higher frequencies of the overall physical interactions with the screen. The active group was expected to utilize two functions of tapping: selecting what to learn during learning and providing correct answers during testing. On the other hand, the passive group use tapping mainly to choose their answers during testing. This difference might have contributed to different levels of cognitive loads between the two conditions. In other words, interactivity during the familiarization phase might have benefited the passive group through the goldilocks of interactivity – not too much and not too little but just right – to prepare children to choose correct answers. I think the authors should address this point in the manuscript. Although the authors discussed the findings based on cognitive resources available during encoding and prepotent motor responses in the discussion section, it was not clear to me how they interpreted the interactions available for both conditions during the familiarization phase.

- See responses to comment above. We suggest that since we do not find difference in children’s target looking in the active and the passive condition, that it is likely that both children learned the word-object associations equally well. Rather, there appears to be a deficit in showing their successful learning that led to the differences in response time and accuracy data
