# Supplementary material for: Word learning from a tablet app: Toddlers perform better in a passive context
Source: PLoS One. 2020 Dec 1;15(12):e0240519. doi: 10.1371/journal.pone.0240519 (PMC7707543; doi:10.1371/journal.pone.0240519)
Supplement: Attachment — Submitted filename: Ackermann Lo response to reviewers.docx [file pone.0240519.s005.docx]

Experiment 1:

1. You provide the structure of your models which is very helpful but there is no justification for why you decided to omit the random slopes from the models. The random slopes of Age are especially important in the Familiarisation phase were children are presented with real words and their knowledge of these may differ across age groups. If you tried the maximal random effect structure and the models did not converge then please state so explicitly.

Thank you for pointing this out. The earlier model did not include age as a random effect as the initial dataset only included data from one age. However, we updated the model accordingly and ran into convergence issues when we tried to fit a maximal random effects structure, which we have now also reported in the paper.

2. Unfortunately, it is difficult to appreciate the effects of Condition and Age because your figures do not correspond to the models as they report the data split by Trial Number. As a result, the main effects and interactions are not clear from the figures. As requested by Reviewer 1, please provide figures that show the aggregate effect of Condition and Age for each experimental stage and dependent variable – a format that depicts not just measures of central tendency and variability but also the shape of the distribution such as violin or raincloud plots would be most informative but this is merely a suggestion. When addressing the dynamics of performance over time in your text (e.g. by referring to early vs. later trials) the current figures can be referred to in an Appendix or Supplementary Materials.

Thank you for the suggestion to include figures that depict the distribution of data. We have now replaced Fig. 3 with a violin plot that shows children’s reaction times in each test phase, split by age and condition. Similarly, Fig. 4 has been replaced by a plot showing the mean accuracy for each age group and test phase. The plots by trial have been moved to an appendix.

3. My last point is potentially serious unless I misunderstood your reporting (in which case I apologise): I wasn’t sure whether you are reporting the correct reference category. For example, in the RT model, if in the Familiarity phase children in the Active condition were faster as you suggest in line 228, and Passive was the reference condition, I would expect the slope estimate (beta) to be negative. Similarly, for 2AFC RTs, if passive children were faster and Passive is the reference category then beta should be positive if the active children were slower. A similar discrepancy arises in the Accuracy analyses. Please check whether the reference category corresponds to the reported direction of effects. Also, please clarify whether you are using sum or treatmen t coding as this has implications for interpreting the intercept. My big concern is that if Passive was indeed the reference category then your models show exactly the opposite of what you claim.

Thank you for pointing this out. We have now clarified what coding scheme was used and how this relates to the intercept and the beta estimates. In our sum coding scheme, passive is coded as 1 and active as -1. A positive beta, as in the model for the familiar phase, thus indicates that the condition coded as 1 (passive) has a higher RT than the intercept (mean RT ignoring effect of condition). We opted for sum coding instead of treatment coding as this makes the intercept interpretable as an overall measure of performance regardless of condition.

Experiment 2:

1. Similarly, as requested for Experiment 1, please provide a graph for the learning phase with overall fixation proportions that illustrate the main effect mentioned in lines 443/444, i.e. something similar to Figure 7.

We have now provided a graph for the learning phase.

2. For the mixed-effect models, you state that you used sum-coding. Was sum coding also used for the RT and accuracy analyses in Experiment 1? Please clarify. Again, there is confusion with the estimates: if Passive is the reference category then a positive beta for Condition (e.g. 0.219 for 2AFC) would suggest accuracy is higher in the active group? This is not what Figure 10 suggests so is it possible that Active was the reference category? Again, this may have serious implications for your results so I hope that your reporting of the reference category was just a simple error and Active is the reference category (this is likely because lme4 takes the category that comes first in the alphabet as reference category unless specified otherwise). Please check and clarify.

Thank you for pointing this out. See our response above. We have now clarified this in the paper.

6. Review Comments to the Author

Reviewer #1:

What remains unclear to me is :
(1) how the authors decided on how many participants to add to Experiment 1

Thank you for this question. We had initially decided to test 20 children in each condition in each age-group (i.e., 40 children in each age-group). However, due to an error, we ended up testing 22 children in one condition in one age-group. We, therefore, increased samples across age-groups to test 22 children in each age-group. Posthoc power analyses revealed that this was adequate for our main tests.

(2) why Experiment 2 was specifically conducted with Malay-speaking children

This was a convenience sample given that two of the authors were located in Malaysia at the time of data collection. Indeed, this was the same reason that we tested German children in the initially submitted study. We do not place any value on the origin of the children tested in the study, in keeping with the criticism of the WEIRD focus in the literature, which does not typically require similar justification of why predominantly Western Educated children are tested in most studies in the field. We merely wish to highlight with a different sample of children that we replicate the results in children from two different cultural and language backgrounds and hope by this to contribute to the diversity of the findings reported. We have now made this clear in the article.

(3) how might the reported experiments contribute to the discussion on the effects of children’s choice of what they learn on learning outcomes (cf Sim et al.) and what the developmental trajectory of these effects might look like

We thank the reviewer for pointing us to this paper. This is a very interesting study, which, nevertheless is at a tangent to the current paper. In this study, the authors create, to a certain extent, an information gap which can be resolved by children in their active selection of objects that could close this information gap. Similar work with children in a word learning study (Zettersten & Saffran, 2019) finds that children actively choose objects that reduce the information gap, similar to Sim et al. (2015) who find in a non-word learning paradigm that children sample objects that help them determine the categorisation of objects to different houses. In our study, however, we did not create a similarly imbalanced information gap, at the very least, there was no informational gain, apart from salience or children’s preference for one object over the other, which would bias children to choose one object over the other. Therefore, it is difficult to consider the development of such effects using such disparate paradigms. We have however noted the two studies and their findings and the relation to the current work in the Introduction.

(4) why children’s frequency and duration of lookaways in Experiment 2 wasn’t analyzed: this would have provided us with some insight about the potential differences in children’s engagement with the task and their effects on word learning (if any).

We report as is standard in the literature the proportion of target looking to the images during both the training and the test phases. This is by far the most oft-reported index of children’s performance in the literature and through the plotting of both time course graphs in addition to the overall statistics provides an overview of children’s looking behaviour across the trial. In addition, we note that we also plotted the looking time during the learning phase in Figure 6 of the old manuscript (current Figure X). This captures duration of lookaways during the training phase (since the proportion of target looks would drop during at any given time if the child were looking away) and showed a difference between the active and the passive children (in the timewindow between 7200 to 9800ms). We also note that the proportion of target looking in the learning phase was not a significant predictor of participants’ responding in the trial. Given this analyses and the established measure of proportion of target looking that we do report, we did not add a further variable(s) to the analysis.

I suggest that the authors address these questions in the next round of revision.

Other points:
Line 24: please clarify what you mean by “passive in such situations”.

Done.

Line 54: The transition to talking about the video deficit is abrupt. Screen media does not necessarily imply *passive viewing* in the context of tablets. Consider adding an opening sentence that will orient the reader and help expect the discussion about the video deficit.

Done. We have now made clear that in this context, we refer to a video deficit when children were exposed to training stimuli on screen passively (and explained what this may mean).
Line 64: Define pseudo-social. I can infer from my previous comments what this means, but the reader will need an explicit definition.
Done.

Lines 71-73: Specify the ages of “younger toddlers” and “older toddlers”.
Done.
Table 1: Consider renaming 36-mo group a “40-mo group”. The average age is 39.62 months.

We agree and have changed this throughout the manuscript

Line 163: Perhaps child-directed speech? Infant-directed speech seems strange here.
Done

Line 193: Remove superscript from “was”.
Done

Table 2: Display mean reaction times separately by condition.

Done

Lines 269-271: I am having trouble parsing this sentence. Please consider rephrasing. Something like “In this experiment we set out to examine whether the opportunity to choose the objects that will be labeled influences children’s learning of those labels”.

Done. We have now changed it as follows: In this experiment, we set out to examine whether being given the opportunity to choose the objects that will be labeled influences children’s learning of these word-object associations in a touchscreen-based word learning task.

Line 288: So, this effect seems to be pretty small and relatively hard to interpret? Perhaps discuss if anything can be made of this? There was a discussion in the intro about the effects of age, which, I believe, should continue in the results section of Experiment 1. Does the observed condition*age interaction inform our understanding of the developmental trajectory of children’s ability to benefit from choosing what they learn?

First, we now make clearer that differences in the familiar test phase are to be treated with caution for the reasons mentioned earlier in the same paragraph. Second, we now make clear that collapsing across the measures we tested, there are no developmental differences in the passive benefit that we find at all ages. Thus, this was a response time boost at the younger age-group and an accuracy boost at the older age group. But in both cases, this was a passive benefit and not as hypothesised an early active benefit changing to a passive benefit.

Line 296: Remove the hyphen from age groups.
We have done this throughout the manuscript.

Line 385: Remove “both”.
Done

Lines 406-431: It seems to me that considering that the level of engagement with the app could be a potential factor that influences children’s learning, it would be reasonable to analyze the frequency and the duration of lookaways in each condition. Looking to the target object during the learning phase does tap into this question but it misses out on children’s overall level of engagement with the task.

As we note above, the proportional of target looking throughout the learning phase was coded and entered into the model during target recognition and was not a significant predictor of children’s target recognition success. This provides us with children’s engagement during the training phase and suggests that there was no evidence that engagement during the training phase predicts learning. Furthermore, we plot the proportion of target looking during the test trials and the learning trials. This time course captures the duration of lookaways since the proportion of target looking will systematically drop were children to look away at any given point in the trial. We did not examine frequency and duration of lookaways given the established measure that we incorporate into the analyses, namely, the proportion of target looking measure.

Lines 619-630: Unclear to me if this is quite the right conclusion. If children learn regardless of the condition, it suggests that the level of app interactivity (at least as far as controlling the information presented) is not a predictor of learning outcomes. The way I see it, this is not a good reason for parents or educators to be alarmed.
We have now rephrased this to highlight that the apps may not be accurately tapping into children’s learning progress (as was seen in the case of the active children) depending on how it is structured.

Reviewer #2:

Page 13: In the discussion section of Experiment 1, the authors said, "there were no differences in children’s accuracy of responses" (Page 13, L277-278) during the familiar test phase. However, the results section of Experiment 1 includes the following: "the familiar phase and the 2-AFC, condition significantly predicts accuracy, with children providing more accurate responses in the passive condition relative to the active condition" (Page 13, L260-261). These two statements contradict each other, so it should be corrected.

 Thank you for pointing this out. We have now corrected this.
