# Supplementary material for: Word learning from a tablet app: Toddlers perform better in a passive context
Source: PLoS One. 2020 Dec 1;15(12):e0240519. doi: 10.1371/journal.pone.0240519 (PMC7707543; doi:10.1371/journal.pone.0240519)
Supplement: Attachment — Submitted filename: Rebuttal_Ackermann_Lo 2020-09-25.docx [file pone.0240519.s006.docx]

**Rebuttal letter: PONE-D-19-23077R2
Word learning from a tablet app: Toddlers perform better in a passive context**

Dear Dr. Kampe,

We would like to thank you for your insightful comments. We enclose below a detailed point-per-point list of changes made in response to your comments. Once again, we would like to thank you for contributing to a marked improvement of the manuscript and we hope that the new version addresses adequately your concerns.

Sincerely,

The Authors

---

**Comment 1:** The wording from line 233 onwards (and the equivalent for Exp 2) should be rephrased to explain why there was no random slope of Age by Words. You presently refer to a random effect of Age which is not quite correct as you have random effects of Participant and Word. In essence, you should explain why your model did not contain the full random effect structure

RT_log_ ~ Condition * Age + (1|Participant) + (Condition * Age|Word)

I am not suggesting you change the model but am asking you to state clearly that full random effect structure with slopes of Condition, Age and their interaction by Word resulted in convergence problems. This is just a request to put the wording right.

**Response 1**: Note that age was entered into the model as a fixed effect (as mentioned in line 234), not as a random effect. We examine performance here on a 2-AFC and a 4-task here where the object is the target in one trial and the distractor is the other. Therefore, performance on a given word cannot be entirely attributed to the word alone but also the distractor displayed in test trials. Thus, including variation across individual words in the effect of condition*age is confounded by influence of the distractor in each trial. We tried initially to reduce this confound by not running the maximal model. We have now, however, corrected this the following way. We now run models with random effects on the object rather than the word and focus on the leaner models in the manuscript while reporting the results of the full maximal models in Appendix C. We also note that this was our reason for not reporting the maximal models in the paper on page XX. There were a few minor deviations across the lean models and the maximal models which we highlight across the manuscript. In particular, the accuracy results in the 2-AFC and 4-AFC task remain the same across all studies, while differences in the reaction time measure at 24-months disappear.

Note that the model we run differs from the model suggested by you since we examine the data by pairs (i.e., the active-passive pair) and could therefore include variation in the effect of condition across the random effect of participant pairs. Running the models as suggested by you with participants rather than pairs yields very similar results, but given the design of the study we retain the paired analyses. Finally, in rerunning the analyses we realised that 1 pair in the German study at 24-months and 3 pairs in the Malaysian study were not age-matched and therefore have excluded these children from the new analyses. We apologise for not noticing this sooner.

**Comment 2**: I also note that in figures A1 and A2 the group now labelled as 40-months-old is still labelled as 36-months-old – please correct this. Also, you currently have two figures 2 – I presume the latter figure labelled 2 on the top left of the page is meant to be figure A2. While you are at this it would help if you could put the figures in the right order to expedite the final editing process.

**Response 2:** Many thanks for pointing this out. We have now relabeled both figures and re-ordered the figures.

**Comment 3**: Line 240: Please change ‘The model included the interaction…’ to ‘The models included the interaction…’ as you are describing three models, one for each task.

**Response 3:** Many thanks for pointing this out. We have now updated the sentence accordingly.

**Comment 4:** I am still somewhat confused about the coding of the effect of Age in the analyses of Experiment 1. The sum-coding of Age adopted now makes it difficult to interpret the coefficients and to ascertain whether performance in the 30-months-olds differed from the 24-months-olds, and whether performance of the 40-months-olds differed from the 30-months-olds. My preference would be for treatment coding with either 24- or 30-months-olds as the reference category but to improve general interpretation of the model results please try to be a bit more explicit in the text regarding the effects of Age in the Familiarisation and 2-AFC tasks.

**Response 4:** Thank you for pointing this out. We have now explicitly clarified the interpretation of the models as follows:

RT (Table 3): “In other words, there was a difference in the effect of condition between 24- and 30-months and between 24- and 40-months in the 2-AFC model, but not in the familiar or the 4-AFC model.”

Accuracy (Table 4): “ There was a difference in the effect of condition between 24- and 40-months in the familiar model and between 24- and 30-months in the 2-AFC model. There was no effect of condition on accuracy in the 4-AFC task. There were no interactions between condition and age between any of the other ages tested in any of the other models.”

**Comment 5:** In your narrative that explains the RT analyses on pages 11/12 please include a brief summary of the model results for the 4-AFC task – Table 3 shows there were no significant effects but that should be stated explicitly in the text.

**Response 5.** Thank you for pointing this out. We have now explicitly stated this in the text as follows: “Furthermore, there was no effect of condition in the 4-AFC task, nor were there any interactions between condition and age in this task (see Table 3).”

**Comment 6:** Please tone down the interpretation in lines 303-305 – while the 24-months-olds were indeed the only ones showing an effect of Condition it is misplaced to call it “much faster” based on what the violin plot in Figure 3 shows. “Somewhat faster” seems more appropriate.

**Response 6:** Many thanks for pointing this out. We have now updated the sentence accordingly.

**Comment 7:** Please label the dashed horizontal line in Figures 6 and 7.

**Response 7**: Both figures are now updated.

**Comment 8:** Lines 310 and 314 referring to the ‘passive benefit across the ages’ sound redundant – please rephrase.

**Response 8:** Many thanks for pointing this out. We have now removed this sentence.

**Comment 9:** To address the points raised by Reviewer 1, please include a brief justification for analysing only looks to the target and not lookaways. You have justified it in your rebuttal letter but other readers may have similar questions so it would be good to address this head-on, somewhere in the vicinity of lines 406-409.

**Comment 9:** We have now included the following sentence where suggested by you. “We report, as is standard in the literature, the proportion of target looking to the images during both the training and the test phases. In addition, we plot the looking time during the learning phase and the test phase in Figures 6, 7 and 8. Together these capture not just the proportion of looks to the target but also the look-aways to the distractor since the proportion of target looks would correspondingly drop at any given time were the child to be looking at the distractor than the target.”
